# Estrogen and androgen receptor expression in surface epithelium and inclusion cyst in the ovary of premenopausal and postmenopausal women

**DOI:** 10.1186/1757-2215-6-85

**Published:** 2013-11-27

**Authors:** Carmen Mendez, Flavia Morales-Vasquez, Delia Perez-Montiel, Maria J Gomora, Clementina Espinola-Zetina, Azucena Hernandez-Martinez, Horacio Lopez-Basave, Enrique Pedernera

**Affiliations:** 1 Departamento de Embriologia, Facultad de Medicina, Universidad Nacional Autonoma de Mexico, Ciudad Universitaria, 04510, Mexico, DF, Mexico; 2 FMV Departamento de Oncologia Medica, Instituto Nacional de Cancerologia, DPM Departamento de Anatomia Patologica, HLB Departamento de Cirugia Oncologica, Mexico, DF, Mexico; 3 Hospital Militar de Especialidades de la Mujer y Neonatologia. Secretaria de la Defensa Nacional, Mexico, DF, Mexico

**Keywords:** Epithelial inclusion cysts, Ovarian surface epithelium, Human ovary, Estrogen receptor, Androgen receptor, Menopause, Cervical carcinoma

## Abstract

**Background:**

The importance of surface epithelium and epithelial inclusion cysts in the ovary arises from studies demonstrating that these structures are susceptible to epithelial ovarian cancer development. The expression of estrogen receptor alpha (ER alpha), androgen receptor (AR), in epithelial cells of the ovary from premenopausal and postmenopausal women is interesting because sexual steroid hormones are involved in cell growth and differentiation.

**Methods:**

The presence of ER alpha, AR, and the orphan G protein-coupled receptor 30 (GPR30) was demonstrated by immunofluorescence in ovaries obtained from 79 pre and postmenopausal patients, undergoing histero-salpingo-oophorectomy for proliferative gynecological diseases. The proportion of patients that displayed positive reaction for estrogen and androgen receptors in epithelial cells of the ovary was evaluated according to menopausal status and associated pathology.

**Results:**

The proportion of patients that displayed a positive receptor expression in the epithelial cells of the ovarian surface and cortical inclusion cysts shows that ER alpha is present in 20 of 79 patients (0.25), AR in 33 of 79 (0.42) and GPR30 in 38 of 55 (0.69). There are no differences in ER alpha, AR, and GPR30 expression between pre and postmenopausal patients and considering the associated pathology, proportions for ER alpha and GPR30 are similar. The patients with cervical cancer show a higher proportion of AR expression in epithelial cells of the ovary, which is statistically significant (P < 0.01) compared with patients with other proliferative diseases.

**Conclusions:**

The presence of ER alpha, AR, and GPR30 in the surface epithelial ovarian cells and its derivatives are observed with a proportion that is specific for each receptor. The proportion of expression for these receptors in the epithelial cells of the ovary does not change after menopause. The proportion of ovaries with AR positive epithelial cells in patients with cervical squamous carcinoma is higher compared with other gynecological pathologies.

## Introduction

The human ovary presents important changes after the fourth decade of life; the number of follicles that are recruited increases in the menopausal transition, the production of estrogens is erratic and the level of progesterone is diminished [1-3]. The follicular reserve is markedly reduced at menopause, the ovary is devoid of growing follicles and estradiol secretion is diminished; meanwhile, testosterone levels are maintained, at least at early postmenopause [4,5]. The ovary at postmenopause is characterized by a reduced size with an irregular surface displaying invaginations. An atrophic cortex without follicles is replaced by a fibrous stroma covered by the surface epithelium that is also found in surface clefts. Epithelial inclusion cysts could be visualized in the cortical region; the origin of these inclusion cysts in the ovary has been related to invaginations of the surface epithelium or to ruptures of the surface epithelium during ovulation [6]. Alternatively, epithelial cells from the Fallopian tubes may originate inclusion cysts after being implanted into the ovary, as suggested by the occasional presence of ciliated and secretory cells in cortical cyst [7]. The importance of surface epithelium and epithelial inclusion cysts arises from studies demonstrating that these structures eventually presented dysplastic precursor lesions and are susceptible to develop epithelial ovarian cancer [8,9].

Steroid hormones interacting with their receptors regulate several cellular events, such as differentiation, hypertrophy and hyperplasia, modulating the transcription of specific genes. The surface epithelium and cortical inclusion cyst are exposed to changes in the hormonal environment of the ovary, mainly in the perimenopausal and early postmenopausal period. Moreover, the interaction of the epithelium, surrounding stroma and steroid hormones would be important to maintain the epithelial morphology and even in the development of metaplasia and dysplasia processes. A previous study reported that ovarian surface epithelium expressed estrogen receptors (ER alpha and ER beta), androgen receptor and progesterone receptor in primary cultures obtained from postmenopausal women [10]. The presence of ER alpha has been demonstrated by immunohistochemistry in postmenopausal women in the ovarian surface epithelium and in epithelial inclusion cyst [11]. Similarly, AR has been detected in the female reproductive tract [12], including the surface epithelium and cortical inclusion cyst of the ovary [11,13].

On the other hand, the orphan G protein-coupled receptor 30 (GPR30) has been proposed to mediate non-genomic action of estrogens, through the activation of the epidermal growth factor receptor pathway, inducing the expression of factors related to the progress of the cell cycle in ovarian cancer cells [14,15]. To our knowledge, the presence of GPR30 in epithelial structures of the human ovary has not been described.

The purpose of this study was to evaluate the presence of ER alpha, AR, and GPR30 in the ovarian surface epithelium and epithelial inclusion cysts in apparently normal ovaries from premenopausal and postmenopausal women.

## Materials and methods

### Human tissues

Ovary samples were obtained from 79 patients undergoing hysterectomy and salpingo-oophorectomy for proliferative gynecological diseases as approved by the Instituto Nacional de Cancerologia, México DF. The buffered formalin fixed samples were obtained from the Pathology department during 2009–2012 and processed for demonstrating the presence of ER alpha, AR, and GPR30 by immunofluorescence. The protocol of the study was approved by the Ethical Board of the Instituto Nacional de Cancerologia and Facultad de Medicina, Universidad Nacional Autonoma de México.

### Patients

Patients between 32 and 82 years of age were studied, of which only 7/79 have less than 40, according to clinical diagnosis 54 patients were postmenopausal and 25 premenopausal. Premenopause refers to the whole reproductive period prior to the menopause. Postmenopause is defined as 12 consecutive months of amenorrhea; patients with induced menopause by other pathologies were not included. Antecedents of pregnancy were reported in 70 patients and 66 refused hormonal contraception. According to the indication of oophorectomy, patients had been previously diagnosed with cervical squamous carcinoma (n = 28), endometrial adenocarcinoma (n = 28), uterine leiomyoma (n = 10) and other less frequent pathologies (n = 13).

### Immunofluorescence

The formalin fixed samples were paraffin embedded, sectioned at 5 μm thickness and placed on coated glass slides (Biocare Medical). Slides were kept at 56°C overnight, sections were deparaffinized by incubation in xilol during 15 minutes, then, rehydrated through a graded series of ethanol and water. After hydratation, samples were exposed to antigen retrieval with 1X Diva Decloaker (Biocare Medical) in a pressure cooker for 10 minutes. Slides were incubated overnight at 4°C with the following polyclonal rabbit primary antibodies: anti-AR 1:50 (Santa Cruz Biotechnology, Inc.), anti-ER alpha 1:50 (Santa Cruz Biotechnology, Inc.) and anti-GPR30 1:600 (ab39742, Abcam antibodies) and further incubated with a secondary antibody goat-anti-rabbit Cy3 (AP187C, Millipore). In order to identify epithelial cells, the slides were secondarily incubated overnight at 4°C, with mouse monoclonal anti pan-cytokeratin AE1/AE3 + 8/18 (CM162C) diluted 1:100, Biocare Medical). The samples were washed with PBS and incubated with the secondary antibody Alexa Fluor 647 donkey anti-mouse (A31571), (Invitrogen). Nucleus was stained with 4′, 6-Diamidino-2-Phenylindole, Dihydrochloride (DAPI) (Sigma Chemical). Slides were mounted with VectaShield mounting medium (Vector Laboratories). The immunolabeled slices were observed using a confocal laser microscope Leica TCS SP5 (Leica Microsystems, Wetzlar, Germany). Negative controls involved the substitution of the primary antibody with either PBS or with pre-immune immunoglobulins; for AR and ER alpha, primary antibodies were incubated with a blocking peptide. Positive control tissues were included in each immune reaction. The epithelial cells present in the surface of the ovary, in epithelial clefts connected to the surface, and epithelial cells of cortical inclusion cysts were evaluated; follicular cysts were not included. The presence of labeled epithelial cells in any of these locations with an H-score ≥ 50 was considered positive. Positive reaction for ER alpha and AR was detected through nuclear staining, for GPR30 the labeling was observed in the cytoplasm. The classification of double blinded samples was assessed by two independent observers.

### Statistical methods

Frequency of positive reactions for each receptor was evaluated for association (chi-square) and comparison of proportions (“Z” value for normal distribution). P value less than 0.05 was considered significant.

## Results

**Figure 1:**
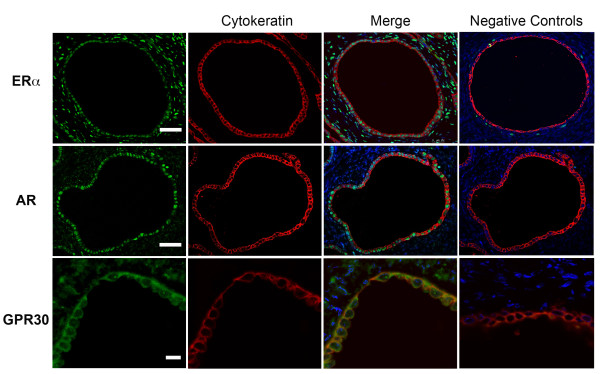
**Immunofluorescence for ER alpha, AR, and GPR30 in ovarian epithelial cells of postmenopausal women.** ER alpha, AR (green) are visualized in nuclei of epithelium of cortical cyst, GPR30 is observed in the cytoplasm of the ovarian surface epithelium. Cytokeratin (red) indicates epithelial cells; DAPI (blue) stains the nucleus as displayed in merge images. Scale bars indicate 50 μm for AR and ER alpha and 10 μm for GRP30.

Positive reactions for ER alpha, AR y GPR30 were observed in epithelium of ovary sections; co-localization with cytokeratin allowed the identification of epithelial cells in the ovarian surface and cortical cysts. Nuclear staining with DAPI indicated the ER alpha and AR nuclear localization (Figure 1).

The presence of ER alpha was observed in 0.25 of the total samples; the proportion of AR positive samples was 0.42; while GPR30 was observed in 0.69 of the samples. There was no difference in proportions between premenopausal and postmenopausal women (Table 1).

**Table 1:** Expression of ER alpha, AR and GPR30 in ovarian epithelial cells in pre and postmenopausal patients

	**ER alpha**	**AR**	**GPR30**
Premenopause	7/25	8/25	12/21
	**0.28**	**0.32**	**0.57**
Postmenopause	13/54	25/54	26/34
	**0.24**	**0.46**	**0.77**
Total	20/79	33/79	38/55
	**0.25**	**0.42**	**0.69**

Positive reactions for ER alpha, AR, and GPR30 in epithelial cells of cortical inclusion cyst and positive cells in the surface epithelium or epithelial clefts in the same sample were compared. Relationship of positive labeling in both locations has been observed in 78% of ovaries. On the other hand, the association between ER alpha, AR, and GPR30 positive reactions in the same sample was not significant.

Proportions of ER alpha, AR, and GPR30 immunoreactive epithelial cells in the ovary of patients with cervical squamous carcinoma versus patients with other pathologies were compared. There were no differences in the expression of ER alpha and GPR30 between the two groups of patients. The presence of AR in the ovary was significantly higher in the cervical cancer patients than other pathologies analyzed (0.64 vs. 0.29) (Table 2). This increment in the proportion of AR was observed in the ovary of premenopausal and postmenopausal women with similar values.

**Table 2:** Expression of ER alpha, AR and GPR30 in ovarian epithelial cells according with associate pathology

	**ER alpha**	**AR**	**GPR30**
Cervical cancer	9/28	18/28*	16/23
	**0.32**	**0.64**	**0.69**
Endometrial adenocarcinoma, leiomyoma and others	11/51	15/51	22/32
	**0.22**	**0.29**	**0.68**

*P < 0.01 vs. Endometrial adenocarcinoma, leiomyoma and others. Z value = 3.01.

## Discussion

The detection of immunoreactivity by confocal microscopy is a reliable method to evaluate colocalization; moreover, immunofluorescence also prevents changes in the labeling of epithelial cells in relation to the exposure time, as could be observed in techniques based on enzymatic reactions.

In our study, not all ovarian samples were positive for ER alpha, AR, and GPR30 in epithelial cells from the surface epithelium or the cortical inclusion cysts. The immunoreactivity for ER alpha is the least frequent in ovary samples, only a quarter of the ovaries were positive. The presence of AR and GPR30 was observed in about the half of the ovaries, 0.42 and 0.69, respectively. Previous studies have shown the expression of the mRNA for ER alpha, AR, and PR in four primary cultures of surface epithelial cells obtained from ovaries of postmenopausal women [10]. Moreover, the expression of AR, PR, and ER alpha has also been described in cells of the surface epithelium and epithelial inclusion cysts of ovaries of postmenopausal women; the expression of AR and PR remain unchanged, but intensity for ER alpha decreases in relation to years after menopause [11]. Our results do not detect changes in the frequency of positive reaction for ER alpha, AR, and GPR30 in the ovary of premenopausal and postmenopausal women; this discrepancy could be explained because the present study evaluates proportion of positive reaction observed in the ovary, without discriminating the intensity of reactions.

The expression of the hormone receptors is detected in epithelial cells of the ovarian surface and epithelial inclusion cysts; however, in about 20% of ovaries, the positive reaction does not coincide in both locations. This finding suggests that cortical inclusion cysts could be a heterogeneous structure, as it has been proposed in relation to the metaplasic changes detected in the epithelium of inclusion cysts [16]. However, changes caused by the tissue processing techniques could not be totally discarded.

The increase in the proportion of AR positive immunoreactivity in epithelial cells of the ovary in women with cervical squamous carcinoma is an unexpected finding. It seems to be specific because the proportion of ER alpha and GPR30 is similar in patients with cervical carcinoma compared with other proliferative pathologies.

The presence of androgen receptor in the epithelial cells of the ovary could represent the constitutive expression of this receptor in female reproductive tissues. Then, the increase in the frequency of AR that we observed in epithelial cells of the ovary would be associated to changes in androgen receptor expression in cervix. A previous study indicates that androgen receptor expression changes in invasive squamous cell carcinoma of the cervix compared with normal cervical epithelium [17]. Moreover, AR regulates the expression of key factors involved in cell proliferation and invasion, as cyclin D1, TGFbeta1 and E-cadherin [18]. Therefore, extensive studies will be required to corroborate the relationship of cervical carcinoma with androgen receptor expression.

Alternatively, human papillomavirus (HPV) infection could simultaneously affect cervix and other reproductive organs. There is evidence of the presence of HPV (types 16, 18, 31, 33, 53) in the human ovary [19] and HPV transcripts were detected in uterine endometrioid carcinoma [20]. Changes in the expression of androgen receptor in the ovary would be related to HPV infection. Similar to previous findings in cervix describing a high estrogen receptor expression related with HPV infection; this observation is not dependant on a particular HPV type [21]. Future studies will address the relationship of HPV infection with AR expression in epithelial cells of the ovary.

## Conclusions

The expression of ER alpha, AR, and GPR30 should be considered within the phenotype of the surface epithelial ovarian cells and its derivatives. The proportion of positive expression for these receptors in the epithelial cells of the ovary does not change after menopause. The proportion of ovaries with AR positive epithelial cells is highest in patients with cervical squamous carcinoma compared with other gynecological pathologies.

## Competing interests

The authors declare that they have no competing interests.

## Authors’ contribution

EPA, CMH, experimental design, results analysis, manuscript writing, FMV, HLB patients selection. CEZ, AHM, DP, pathology diagnosis, analysis of results, manuscript review. MJG tissue processing and immunofluorescence techniques. All authors participated in discussion of results, read and approved the final manuscript.
